# Nanomaterials as Delivery Vehicles and Components of New Strategies to Combat Bacterial Infections: Advantages and Limitations

**DOI:** 10.3390/microorganisms7090356

**Published:** 2019-09-16

**Authors:** Atanu Naskar, Kwang-sun Kim

**Affiliations:** Department of Chemistry and Chemistry Institute for Functional Materials, Pusan National University, Busan 46241, Korea

**Keywords:** multidrug resistant bacteria, antibiotic resistance, nanomaterials, antibiotic alternatives, nanoparticles, biocompatibility, functionalization

## Abstract

Life-threatening bacterial infections have been well-controlled by antibiotic therapies and this approach has greatly improved the health and lifespan of human beings. However, the rapid and worldwide emergence of multidrug resistant (MDR) bacteria has forced researchers to find alternative treatments for MDR infections as MDR bacteria can sometimes resist all the present day antibiotic therapies. In this respect, nanomaterials have emerged as innovative antimicrobial agents that can be a potential solution against MDR bacteria. The present review discusses the advantages of nanomaterials as potential medical means and carriers of antibacterial activity, the types of nanomaterials used for antibacterial agents, strategies to tackle toxicity of nanomaterials for clinical applications, and limitations which need extensive studies to overcome. The current progress of using different types of nanomaterials, including new emerging strategies for the single purpose of combating bacterial infections, is also discussed in detail.

## 1. Introduction

Antibiotic resistance is described as “the silent tsunami facing modern medicine”, as human beings are reluctantly moving towards the post-antibiotic era where the morbidity and mortality of human beings being dependent on a simple bacterial infection can be a real possibility [1]. The grim impact of antibiotic resistance of multidrug resistant (MDR) bacteria can be recognized by the prediction that MDR pathogens could lead to 10 million annual deaths by 2050 (which at the present time are more than cancer), without immediate intervention [2]. The World Health Organization (WHO) and Centers for Disease Control and Prevention (CDC) have expressed serious concerns regarding this turbulence in medical science. For example, a recent report suggested that among *Staphylococcus* (*S.*) *aureus* strains collected from US hospitals, 40–60% are resistant to methicillin, and these are known as methicillin-resistant *S. aureus* (MRSA) strains [3]. Notably, the more unpleasant situation at this point of time is that in some cases MRSA strains cannot be treated with vancomycin, a supposedly last-resort antibiotic against these strains, and have even resulted in the production of vancomycin-resistant *S. aureus* (VRSA) strains.

In addition to MRSAs and VRSAs, WHO published a catalogue of 12 families of priority pathogenic bacteria that pose the greatest threat to the clinical treatment of infections in human beings [4]. The bacteria are divided into three categories based on the urgency of the need for new antibiotics: critical [*Acinetobacter* (*A.*), *Pseudomonas* (*P.*), and various *Enterobacteriaceae*], high (*Enterococcus faecium*, *S. aureus*, *Helicobacter pylori*, *Campylobacter spp*., *Salmonellae*, and *Neisseria gonorrhoeae*) and medium (*Streptococcus pneumoniae*, *Haemophilus influenzae*, and *Shigella spp*.) priority. Unfortunately, the growth rate of resistance by the above-mentioned bacteria is far more than the advancement of new antibiotics [5]. Therefore, it is imperative to say that alternative antibacterial materials to traditional antibiotics targeting the priority list of pathogens are urgently needed.

Many new alternative approaches to conventional treatment regimens such as bacteriophage [6], antibacterial antibodies [7], combinations of antibiotics [8], photothermal therapy [9], and nanomaterials [3] have been tested. Among such alternatives, nanomaterials are gaining increasing attention from researchers because of their favorable physiochemical properties required for antibacterial activity. Nanomaterials can be defined as the materials that have at least one dimension in the nano range (1–100 nm), whereas nanoparticles (NPs) are particles with at least one dimension in the nano range and can be as small as 0.2 nm. Antibacterial activity of traditional antibiotics is based on hindering and interfering with bacterial cell wall synthesis and intracellular components such as proteins, DNAs, and RNAs. However, bacteria have evolved themselves over the years by mutation and transfer of DNA to diminish the threat posed to them by antibiotics. In this scenario, one of the major advantages of nanomaterials for antibacterial activity is their multi-targeted approach compared to the unidirectional approach of antibiotics [10]. There are five features of nanomaterials that make them a possible alternative to antibiotics. First, nanomaterials can easily penetrate the bacterial cell membrane and damage its structure, resulting in bacterial cell death [11]. Second, suggested mechanisms of antibacterial activity of nanomaterials are similar to the action of antibiotics, including reactive oxygen species (ROS)-mediated oxidative stress, cell membrane disruption, intracellular protein synthesis inhibition, and leakage of intracellular components [12]. ROS mainly include superoxide (O_2_•^−^), hydroxyl radical (^•^OH), singlet oxygen (^1^O_2_), and hydrogen peroxide (H_2_O_2_), and generation of ROS by nanomaterials is regarded as the main reason for nanomaterial-mediated antibacterial activity. Third, various nanomaterials can act as antibiotic drug carriers to help effectively administer antibiotics to their target locations by reducing the probable adverse effects of antibiotics [3]. Fourth, the retention power of nanomaterials in the body is much more than antibiotics, which could be favorable for long-term therapeutic effects [13]. Finally, nanomaterials can be functionalized according to their target and purpose of use as they can be effective against bacterial cells without being toxic against mammalian cells [3].

Nanomaterials include inorganic NPs, graphene-based nanomaterials, black phosphorus (BP), carbon nanotubes (CNTs), and chitosan. There are several reports available which have reviewed alternative therapies to MDR pathogens. Most of them were based on metal oxide NPs [12,14,15] or inorganic NPs [16]. However, not many have explicitly focused on nanomaterials or their associated agents such as inorganic NPs, graphene, CNTs, chitosan, antimicrobial peptides (AMPs), or BP for in-depth discussion of nanomaterial related antibacterial activity. Therefore, in this review we have specifically focused on nanomaterials and nanomaterial-mediated techniques like photothermal therapy, which have shown excellent potential against MDR bacterial cells. Moreover, the advantages of nanomaterials both as antibacterial agents and antibacterial drug carriers are discussed. The strategies to tackle potential toxicity of nanomaterials and some of the limitations along with future perspectives on the relevant research agenda are discussed in this review.

## 2. Advantages of Nanomaterials in Combating MDR Pathogens

The most common working principles of antibiotics are to inhibit bacterial cell wall synthesis, hinder the expression of essential proteins, and to prevent DNA replication [17]. However, bacteria are smart enough to escape this mechanism used by antibiotics. For example, bacteria could modify their cell wall components to prevent the activity of vancomycin, alter their ribosomal structure to acquire resistance to tetracycline, and overexpress enzymes such as β-lactamases and aminoglycosides to degrade antibiotics [3,11]. Besides those strategies, bacteria can develop new resistance mechanisms by modulating their gene expression profiles or receiving vectors from other communicating MDR bacteria. Based on their unique physio-chemical properties, nanomaterials successfully tailor the antibiotic-resistance mechanisms, and therefore can be the possible nano-weapons against the ever-increasing problem of MDR pathogens. The basic difference between nanomaterials and antibiotics is that nanomaterials use a multiple target approach for antibacterial activity rather than the single target approach used by antibiotics [10]. These known multiple targets of nanomaterials are given in Table 1:

Nanomaterials could be successfully tailored to have antibacterial activity without any toxic side effects [13]. Moreover, the administration of antimicrobial agents with the help of nanomaterials would greatly improve the therapeutic index, which in turn extends drug circulation time or extended half-life. Furthermore, controlled drug release can be achieved by functionalization of nanomaterials [13]. Such nanomaterials could help to enhance the activity of antibiotics synergistically. For example, vancomycin-capped Au NPs showed 64-fold improved efficacy against vancomycin-resistant *Enterococci* (VRE) strains and *Escherichia* (*E.*) *coli*, compared to vancomycin alone [18]. Another advantage of nanomaterials is that they can be administered in suitable and cost-effective ways with lowered administration frequency by various routes [19]. Synergistic antibacterial activity, improved solubility, and suspension of drugs are additional advantages of nanomaterials.

Besides their excellent antibacterial properties, nanomaterials can be used as carriers for the delivery of antimicrobial moieties to regions of poor absorption in the body [3]. Several types of nanomaterials including polymer micelles [20], carbon nanomaterials [21], magnetic NPs [22], mesoporous silica NPs [23], polymer-based nanomaterial [24], and dendrimer [25] have already been used as vehicles to carry antimicrobial drugs. The advantages of nanomaterials as antimicrobial drug delivery vehicles are given in Table 1 and explained below.

- Controllable size of the nanomaterials helps to design targeted antibiotics. Due to poor membrane transport activity of some antibiotics, their effect against intracellular pathogens is limited [26]. However, drugs loaded with nanomaterials can easily overcome this issue. Due to their nano size (10–100 nm) nanomaterials can efficiently cross the cell membrane by phagocytosis and enter the host cells via endocytosis [27].
- Drug retention time in blood can be improved by using nanomaterial-based drug delivery systems [28].
- Surface chemistry of NP enables nanomaterials to be soluble in blood stream [3].
- Opsonization is another biological barrier where the physiochemical properties of nanomaterials have been used successfully for systematic delivery of antimicrobial drugs [29]. Generally, opsonin proteins in blood quickly bind to the NPs when they enter blood cells and allow macrophages from the mononuclear phagocytic system (MPS) to bind and remove them. Therefore, several strategies such as encapsulation of polyethylene glycol (PEG) [30] or chitosan [31] with NP have been adopted for increasing the circulation and retention time in blood cells by creating hydrophilic moieties on NP surfaces.
- Nanomaterials can protect antibiotics from detrimental chemical reactions and resistance to targeted bacteria. Researchers have proven that some NPs overcome the traditional “efflux” mechanism of bacteria cells, which normally hinder the uptake of antibiotics by the cells [32]. For example, Liu et al. [33] showed that the dendrimers can hinder P-glycoprotein-mediated efflux in the gastrointestinal tract.
- Nanomaterials also help the antibiotics to minimize side effects. For example, vancomycin is a strong Gram-positive bacterial drug, but can be toxic to the ear and kidney [11]. In this respect, Qi et al. [34] showed that the vancomycin-modified mesoporous silica NPs can be designed to target specific pathogenic Gram-positive bacteria and kill them selectively over macrophage-like cells. It also prevents other harmful side effects because nanomaterial-aided antibiotics are able to reach the target site with more specificity than the antibiotic itself. This also enables high dosage at the infection site.

As described, nanomaterials could become the new weapons (nano-weapons) against MDR pathogens with lots of advantages compared to current antibiotic therapies.

## 3. New Antibacterial Nanomaterials on the Block with New Strategies

**Figure 1:**
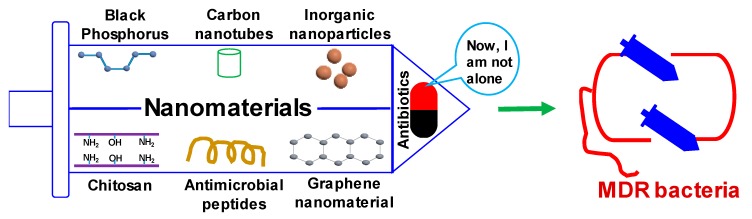
Schematic illustration of combating multidrug resistant (MDR) bacteria with nanomaterials.

Identification of nanomaterials as antibacterial agents complementary to antibiotics has been in full swing. Metals and metal oxides have been widely characterized for their antimicrobial activities. Recently, nanomaterials such as graphene, BP, and polymeric NPs have been used as new weapons to combat MDR bacteria (Figure 1).

### 3.1. Inorganic NPs

#### 3.1.1. Silver Nanoparticles (Ag NPs)

According to recent reports, Ag NPs are the most widely studied nanomaterials for antibacterial activity due to their wide range of activities against various microorganisms [35,36,37]. It is already known that before the discovery of penicillin and other antibiotics, significant research was done on Ag for antibacterial activity. However, the significance of Ag NPs has increased in recent times due to the fact that bacteria rapidly acquire resistance to antibiotics. Silver compounds (metallic silver, silver nitrate, and silver sulfadiazine) are in use for different medical applications such as burn wound treatment, dental work, disinfection of medical devices such as catheters, controlling bacterial infection, and others [38]. Commercially, they can also be used for various textiles, plastic, biopolymer, and coating-based products. The potential for Ag NPs against MDR bacteria has also been reviewed by Allahverdiyev et al. [39]. Moreover, the chances of bacterial resistance to Ag NPs are lower due to their multi-dimensional approaches to express antibacterial activity [40].

Several researchers have suggested different antibacterial mechanisms for Ag NPs. These include damage of the bacterial outer membrane [41], interaction with enzymes, and decomposition of the cellular components [11]. Additionally, the size- and shape-dependent antibacterial activity of Ag NPs has been reported. In this regard, Lu et al. [42] reported that the antibacterial activity is inversely correlated with the particle size of Ag NPs. Among the synthesized Ag NPs of ~5, 15 and 55 nm, ~5 nm Ag NPs showed excellent antibacterial activity. Similar activity was shown by Korshed et al. [43] where the antibacterial activity of laser-generated Ag NPs was inversely correlated with the particle size. In addition to the size, shape-dependent antibacterial activity of Ag NPs has also been reported [44]. Pal et al. [45] reported three different forms of Ag NPs, namely spherical, rod-shaped, and truncated triangular, for antibacterial activity. Among them truncated triangular Ag NPs showed comparatively higher activity due to their high-atomic-density surfaces. Bera et al. [46] also showed similar size- and shape-dependent antibacterial activity against *Staphylococcus* (*S.*) *epidermidis*, *Bacillus* (*B.*) *megaterium*, and *Pseudomonas* (*P.*) *aeruginosa*. They stated that the size and shape of Ag NPs controlled the antibacterial activity and could be used for potential applications such as clinical wound dressing, and bio-adhesives [15].

Another emerging trend in antibacterial researches is the combination of antibiotics with Ag NPs [47]. Recently, Katya et al. [48] showed that the combination of gentamicin and chloramphenicol with Ag NPs is a comparatively better option to treat MDR *Enterococcus faecalis* than antibiotics alone. Additional reports showed that the combination of Ag NPs with antibiotics such as ampicillin, penicillin, amoxicillin, erythromycin, vancomycin, kanamycin, and others increases the original activity of antibiotics [49]. Moreover, functionalized Ag NPs–AMPs expressed synergistic activity in killing bacteria [50]. Therefore, it is worthy to include some clinical research related with Ag NPs to fulfill its potential as an antibacterial agent against MDR pathogens.

#### 3.1.2. Gold Nanoparticles (Au NPs)

Apart from Ag NPs, Au NPs have attracted much attention for their excellent antibacterial activity based on their inert nature, non-toxicity, functionalization with biomolecules, ability to detect bacteria, and photothermal activity [51]. Moreover, the advantage of Au NPs over Ag NPs is that the Au NPs could satisfy the biocompatible nature of physiological cell systems and clinical applications due to their inert nature. Although it is widely accepted that ROS generation is the main underlying mechanism of antibacterial action by nanomaterials and antibiotics, the action mechanism of Au NPs in killing bacteria is performed by additional ways [52]. For instance, the activity of Au NPs was enhanced by electrostatic attractions where the positively charged NPs strongly attached to the negatively charged bacterial cell membrane [53]. Additionally, shape-controlled antibacterial activity of Au NPs has been suggested by Huang et al. [54]. 

Selective or efficient antibacterial activity of Au NPs could be acquired by the modification of the surface. For instance, Mühling et al. [32] showed the selective killing of bacteria by Au NPs after the conjugation of antibodies against protein A with NPs. Such conjugated Au NPs were assembled with laser-induced effects and showed increased damage to *S. aureus* cells. Moreover, several studies have implemented Au NPs along with antibiotics for their synergistic activity to eradicate MDR pathogens [55,56]. For instance, Huang et al. [57] successfully implemented the selective antibacterial activity against MDR pathogens by multifunctional Fe_3_O_4_@Au nanoeggs with the help of photothermal therapy. It is worthy to note that the useful features of Au NPs described above can be further incorporated into clinical usages of antibacterial therapy.

#### 3.1.3. Zinc Oxide Nanoparticles (ZnO NPs)

The increased research on ZnO NPs as antibacterial agents can be attributed to their minimal toxicity to human cells and their selectivity for bacterial cell killing [58]. ZnO NPs have certain advantages over Ag NPs which include UV-blocking property, white appearance, and low cost [59]. For these aforementioned reasons, biocompatible ZnO has been used in several commercial products such as cosmetics, medical equipment, food packaging material, cotton fabrics, and drug carriers [60]. There are several accepted mechanisms of ZnO NPs in killing of bacteria: bacterial cell membrane disruption and leakage of intracellular contents; generation of hydrogen peroxide and Zn^2+^ ions; ROS generation and membrane dysfunction [61,62].

Size-dependent antibacterial action of ZnO NPs has also been reported by several researchers where the activity is inversely correlated to the particle size [51,63]. In this regard, Padmavathy et al. [64] showed that the antibacterial activity of ZnO NPs was increased by decreasing particle size. Furthermore, Hossein-Khani et al. [65] and Emami-Karvani et al. [66] showed that the antibacterial activity of ZnO occurred after reduction in particle size and high powder concentration.

The ROS was generated in dark experimental conditions by fabricating some defects on the surface of metal-substituted ZnO NPs [67]. Such materials showed the ability to inhibit the growth of MDR pathogens in a concentration-dependent manner without expressing toxicity to mammalian cells [67,68]. The promising results of ZnO NPs against bacterial pathogens have prompted researchers to implement ZnO NPs in industrial applications such as food packaging, where evading decomposition and maintaining the color of food are required. Moreover, these ZnO NPs could be used as fungicides in agriculture as they have showed effective antifungal activities against two post-harvest pathogenic fungi *Botrytis cinerea* and *Penicillium expansum* [69]. Therefore, ZnO NPs could be used as potential antibacterial agents against many bacterial pathogens. 

#### 3.1.4. Titanium Dioxide Nanoparticles (TiO_2_ NPs)

Among the reported NPs that have been used as antibacterial materials, TiO_2_ NPs are most widely used as photocatalytic agents in expressing antibacterial activity [70]. This can be attributed to their ability of ROS generation by UV light. However, the use of TiO_2_ NPs under UV light is constrained because the strong light source damages human cells [39]. A possible solution to this issue is doping TiO_2_ NPs with metal ions which shifts the absorption range of TiO_2_ to the visible light range, eliminating the need of UV light irradiation for ROS generation. Doping with metal ions was the most used technique of TiO_2_ NPs for visible light absorption which resulted in increased antibacterial activity [71]. In addition to the above properties, non-toxicity, cost effectiveness, and stability in water make TiO_2_ NPs potential materials for water treatment [72]. Such metal-doping/visible light absorption shifting make TiO_2_ NPs an alternative agent to be used for electric sterilization. ROS-mediated oxidative stress generation and site-specific DNA damage were reportedly the cause of bacterial cell death [73]. Hydroxyl radicals produced by TiO_2_ NPs directly attack the microbial surface of the bacterial cells and damage them, which results in bacterial cell death.

According to latest reports, TiO_2_ can be a promising disinfectant in the food industry and cosmetics due to its ability to kill various microorganisms which are highly resistant to desiccation and UV radiation techniques [74,75]. Due to its antibacterial effect against *Lactobacillus acidophilus*, it can be used in dental implants, toothbrushes, screws, and etc. [76]. Allahverdiyev et al. [39] showed that TiO_2_ NPs was effective against MDR bacteria. Its effect on MDR bacteria such as MRSA was characterized by Roy et al. [73]. According to their report, TiO_2_ NPs conjugated with different antibiotics have successfully inhibited the growth of MRSA. TiO_2_ NPs can also be used for post-harvest disease control. For instance, Maneerat et al. [77] implemented its antifungal activity against *Penicillium expansum* to test post-harvest disease control. The potential of TiO_2_ NPs against MDR *P. aeruginosa* was evaluated by Arora et al. [78]. Exposure of TiO_2_ NPs to UV irradiation seems to enhance the antibacterial activity.

#### 3.1.5. Copper or Copper Oxide (Cu or CuO) NPs

Researchers have shown great interest on Cu NPs as potential antibacterial agents because of their favorable physiochemical properties and low lost [51,79]. Moreover, Cu NPs are easily combined with other agents to enhance the antibacterial activity. For instance, Maruthapandi et al. [80] recently reported enhanced antibacterial activity of polypyrrole@CuO NPs. The antimicrobial activities of Cu-chitosan NPs were also successfully implemented by Usman et al. [81] against several microorganisms such as *B. subtilis*, *P. aeruginosa*, *Salmonella choleraesuis*, *Candida albicans* and MRSAs. Ren et al. [82] showed Cu-chitosan NPs as potential agents for several MDR strains such as epidemic MRSAs (EMRSA-16, EMRSA-15) and MRSA 252. Green synthesis of CuO nanoparticles has also been studied against bacterial cells [83,84]. According to Mahapatra et al. [85], the antibacterial activity of CuO NPs is dependent on the bacterial cell membrane penetration and damage of the vital enzymes in bacteria which in turn influences cell death. Size and concentration-dependent antibacterial activity of Cu NPs was reported by Azam et al. [86]. According to the above reports, we can say that the CuO NPs can be utilized like the other nanomaterials for antibacterial action against different bacterial strains.

#### 3.1.6. Other NPs

Several NPs such as Si, SiO_2_ [15,87], MgO [88], CaO [89], and Al_2_O_3_ [90] have shown significant antibacterial activity. High surface area in nanoscale, low cost, and nanocomposite formation with NPs make SiO_2_ an available option against MDR bacteria, especially for potential clinical nanomaterials to treat oral biofilms [91]. Yamamoto et al. [92] showed that the generation of superoxide on their surface was the major reaction mechanism of antibacterial action by CaO and MgO. The antibacterial mechanism of Al_2_O_3_ is dependent on the interaction between NPs and bacterial cell membrane [93]. Apart from these aforementioned inorganic nanomaterials, some other effective nanomaterials which can be potential nano-weapons against MDR pathogens are discussed in later sections.

### 3.2. Graphene-Based Nanomaterials (GNMs)

In recent years, graphene has become a promising material to tackle MDR bacteria due to its excellent physiochemical properties, biocompatibility, and excellent antibacterial activity. The reason for the increased antibacterial activity of GNMs can be attributed to their high surface area, excellent bactericidal properties, and low toxicity for mammalian cells [94]. Therefore, it is capable of killing the bacterial cells selectively. Different types of GNMs have been tested for their antibacterial activity in recent years. These include graphene, graphene oxide (GO), reduced graphene oxide (rGO), chemically converted graphene, and others [94]. It is also reported that graphene by itself has antibacterial property [95]. Numerous studies have reported the interaction between bacteria and graphene nanosheets, and it was claimed that the ROS-mediated oxidative stress is the only reason behind the antibacterial activity of graphene [94].

The antibacterial activity of Ag-based nanomaterials has been studied during the last 10 years [12,15,46]. One of the major advantages of Ag is that at low concentration it is toxic to bacterial cells but nontoxic to human cells. Meanwhile, the problems of Ag NPs include agglomeration and oxidation in physiological solution. However, this problem can be solved by using graphene for making stable and effective Ag–graphene nanocomposites. A plethora of studies has already been conducted on the synergistic effect of Ag–graphene [94,96]. Similarly, the favorable physiochemical and biocompatible property of graphene has been successfully used for other antibacterial nanomaterials such as Au–graphene [97], ZnO–graphene [69,98], TiO_2_–graphene [99], and polymer–graphene [100]. It is also notable that another alternative approach, namely photothermal effect, to acquire antibacterial activity can be successfully implemented by graphene-based nanomaterials [9]. However, in this scenario, graphene was mainly used with other photothermal components for the heat generation. Ag and Au NPs are mainly used as photothermal components with graphene for photothermal effect-oriented antibacterial activity.

### 3.3. Black Phosphorus (BP)

BP has recently emerged as a new potential antibacterial agent. The layer-dependent wide range bandgap (0.3–2.0 eV) of BP compared to the zero bandgap of graphene makes it an ideal candidate for near-infrared (NIR) light irradiated antibacterial activity [101]. More information related to NIR and its use in clinical application is given in Section 3.7. It also has natural biodegradable properties *in vivo* unlike the much-researched graphene which requires functionalization for biodegradable properties. Furthermore, BP acts both as a supportive substrate and a green reductant for Ag [101] and Au [102] NPs, which are known to be excellent nano-sized antibacterial materials.

BP can generate reactive oxygen species (ROS) and penetrate through the bacterial membrane. The successful combination of BP with other nanomaterials is implemented by the synergistic antibacterial activity of BP-based nanomaterials [102,103,104]. However, the antibacterial mechanism of BP is different from other known pathways of antibiotics, in that, it is mainly regulated by light or ultrasound. Due to the layer-dependent bandgap (0.3–2.0 eV) and NIR light absorption of BP, BP or its composites could be suitable for clinical applications where NIR light is used to kill bacterial cells [101].

Several studies have already been performed to prove the efficacy of BP as an efficient antibacterial agent. The work by Ouyang et al. [101] showed that the Ag@BP nanohybrids can act as a synergistic platform for antibacterial activity against MRSA strains. In the report, BP served its dual role as a reducing agent for Ag NPs and a support for the nanocomposite. NIR light was exposed to use the NIR absorption property of BP for antibacterial activity. Moreover, Wu et al. [103] showed a similar kind of surfactant-free synthesis of Au@BP nanohybrids, where the BP itself acts as a reductant in the synthesis of Au NPs. The Au@BP nanohybrids showed excellent synergistic antibacterial activity. Therefore, it can be suggested that BP is an emerging platform to construct antibacterial nanomaterials to tackle MDR bacteria.

### 3.4. Carbon Nanotubes (CNTs)

CNTs are another type of nanomaterial which can be effective against MDR bacteria. Numerous studies have proved that both single-wall carbon nanotubes (SWCNTs) and multi-wall carbon nanotubes (MWCNTs) are effective against bacterial pathogens [104]. However, SWCNTs seemed to be more effective than MWCNTs and their mechanism was possibly affected by the synergistic combination of membrane and oxidative stress. The advantages of SWCNTs for antibacterial activity are their high chemical stability and ease of functionalization [105].

The antibacterial mechanism of CNTs is influenced by many factors such as diameter, length, and surface chemistry [21]. Kang et al. [106] reported that the single-walled carbon nanotubes (SWCNTs) exhibited more toxicity to bacterial cells compared with multi-walled carbon nanotubes (MWCNTs) because of their smaller size. The diameter of CNTs also plays an important role in toxicity to bacterial cells as the interaction between CNTs and bacterial cells is much easier for shorter diameter CNTs [107]. Therefore, after surveying the toxicity of CNTs to bacterial cells, we can state that the reason for its toxicity depends on membrane disruption ability, ROS generation, and synergistic activity with other antibacterial agents of CNTs [106,107]. The CNTs can also be used for antimicrobial photothermal therapy by utilizing its optical property in the NIR region [108]. However, the successful implementation of CNTs as antibacterial agents still require further research due to some problems regarding their aggregation, stability, and bioavailability. Due to their unique configuration and powerful van der Waals interactions, CNTs are expected to agglomerate in physiological solutions. For example, Wick et al. [109] showed that rope-like CNTs are more cytotoxic than well-dispersed CNTs due to the agglomeration at identical concentrations. Therefore, further research is needed in this regard for the successful implementation of CNTs for antibacterial activity.

### 3.5. Nanomaterials Conjugated with Antimicrobial Peptides (AMPs)

Nanomaterials conjugated with AMPs have showed excellent multifunctional properties and are regarded as promising nanomaterials to combat MDR bacteria. AMPs are short, positively charged, gene-encoded, and ribosome-synthesized polypeptides produced by all living forms such as bacteria, fungi, plants, and animals [110]. AMPs also have broad-spectrum activity against Gram-positive and -negative bacteria, fungi, and parasites [110]. The basic reason for increased research on AMPs as antibacterial agents can be attributed to their interaction with bacterial cells. It is believed that electrostatic interaction between positively charged AMPs and negatively charged bacterial cellular membrane results in increased membrane permeability along with cell lysis [111]. Compared to the action of traditional antibiotics targeting intracellular components, bacterial cells are less likely to be resistant to the electrostatic interaction of AMPs. This makes AMPs better agents in comprising innovative antimicrobial nanomaterials. Till now, several possible antibacterial mechanisms of AMPs such as toroidal model, carpet model, barrel-stave model, and aggregate channel model have been suggested [110].

It is notable that AMPs are known to be effective against both Gram-positive and -negative bacteria. For example, Ye et al. [112] showed that a defensin-like antimicrobial peptide (defensin-NV) can be effective against Gram-positive and -negative bacteria. Similarly, Lin et al. [113] reported that 90% inhibition of biofilm formation by a de novo engineered cationic peptide, WLBU-2, and a natural AMP LL-37 showed only one-third of minimum inhibitory concentration (MIC) of antibiotics such as tobramycin, ciprofloxacin, ceftazidime, and vancomycin. Similar to the *in vitro* antibacterial property, AMPs also showed promising results for *in vivo* antibacterial activity. In this regard, vancomycin, omiganan, telavancin, teicoplanin, daptomycin, and bacitracin are named as possible target antibiotics for clinical use. However, the number of AMPs seeking clinical approval is still quite discouraging. The reason for this low number of clinical AMPs can be attributed to their high toxicity towards mammalian cells [114]. To resolve this issue, several researchers have adopted some modifications to improve the biocompatibility of AMPs in human cells. Structural modification of AMPs by the addition of non-natural amino acids, liposome encapsulation, and peptide cyclization are some of the reported techniques to improve the stability and biocompatibility of AMPs. AMPs conjugated with antibiotics or nanomaterials were also successfully evaluated to see the synergistic antibacterial activity [115]. Considering the potential of AMPs, innovative nanomaterials implemented with antibiotics as potential therapeutic antibacterial agents will be on the rise soon.

### 3.6. Chitosan

Nanoscale chitosan has a wide range of antimicrobial activities against bacteria, viruses, and fungi. The reason for its widespread application for scientific research can be attributed to its biocompatibility, nontoxicity, antibacterial abilities. and ability to act as an absorption enhancer [104,116]. Fernandes et al. [117] reported that the antimicrobial effect of chitosan is strongly dependent on the target bacteria and on the molecular weight (MW) of chitosan. For instance, lower MW chitosan exhibited a higher antimicrobial activity for Gram-negative bacteria (*E. coli*, *Klebsiella pneumonia*, and *P. aeruginosa*), while the reverse happened in the case of Gram-positive bacteria (*S. aureus* and *S. epidermidis*). The synergistic property of chitosan has also been implemented with sulfamethoxazole against drug resistant *P. aeruginosa* [118]. Therefore, it was proved that chitosan-related nanomaterials can be a potential option for MDR bacteria, and it is recommended to use it with antibiotics for possible synergistic antibacterial activity. It is also notable that water-soluble derivatives of chitosan showed a higher antimicrobial activity compared with native chitosan [119].

Moreover, there are various reports available on the antibacterial mechanism of chitosan. One such report states that it is the electrostatic interaction with bacterial membrane, where the positively charged chitosan binds with negatively charged bacterial membrane and increases the permeability of bacterial cell membrane, resulting in leakage of intracellular components and eventually bacterial cell death [120]. It was also suggested that the chitosan inhibits enzyme activities by chelating to trace metals which ultimately inhibit the microbial growth [121]. Finally, it can be said that the nano-scale chitosan could be a viable option for antibacterial activity after successfully implementing its antibacterial mechanism.

### 3.7. Photothermal Effect

Photothermal effect is regarded as another potential antibacterial mechanism against MDR bacterial pathogens (Table 2). Under the irradiation of NIR light, a photosensitizer can be used for high light thermal conversion efficiency [9]. NIR light has the capacity to infiltrate mammalian cells, causing minimal damage to them. Therefore, researchers are now using this NIR light-mediated biomedical application due to its clinical importance. The heat generated from the photothermal effect is now widely used as an alternative to antibiotics for treating bacterial infections. A lot of research on photothermal nanomaterials is happening these days. Graphene [122,123,124,125], CNTs [126], BP [101,103], Au [123,126,127,128], and Ag [101,124,129] are widely researched in this regard, for NIR light-mediated photothermal therapy against MDR bacteria.

Several researches with carbon-based conjugates such as graphene oxide, reduced graphene oxide, and CNTs have already been evaluated successfully. However, in most cases, they are functionalized with some molecules or nanostructures for effective antibacterial solution. This bandgap-related problem of graphene can be solved by BP [101,103]. Due to the thickness-dependent bandgap (0.3–2.0 eV) of BP, it showed excellent optoelectronic properties under NIR irradiation [101]. The potential for BP nanosheets as new antibacterial agents has been evaluated by Ouyang et al. [101], where they showed NIR light irradiated antibacterial activity which had a minimal cytotoxic effect on normal cells. Noble metals such as Ag and Au are also regarded as excellent photothermal agents due to their localized surface plasmon resonance (LSPR) properties [101,102]. In most cases, Au is the preferred choice of researchers regarding metal-based photothermal agents [123,126,127,128]. Copper sulfide (CuS), molybdenum sulfide (MoS_2_), and polyaniline (PANI) are additional nanomaterials which have been tried as photothermal agents after conjugating with some biocompatible polymers [9].

### 3.8. Nanomaterial-Conjugated Antibiotics

Another alternative approach is the combination of nanomaterial with antibiotics (Table 3). Several reports confirmed that the combination of nanomaterials with antibiotics has the potential to combat bacterial resistance [29]. Moreover, dose reduction, and with that the reduction of antibiotic toxicity may also be possible with this functionalization of nanomaterial with antibiotics. Panáček et al. [131] reported synergistic antibacterial activity of Ag NPs with antibiotics against *S. aureus*, *E. coli*, and *P. aeruginosa* at very low concentrations. Franci et al. [132] showed that ampicillin-coupled Ag NPs were able to inhibit the growth of both Gram-positive and -negative bacteria, whereas Ag NPs alone could not do the same. Another nanomaterial, chitosan has been successfully combined with antibiotics to completely eradicate uropathogenic *E. coli* from infected mouse urinary bladders [133]. Combined antibacterial efficacy of graphene with antibiotics was evaluated by Gao et al. [134] to inhibit the growth of *E. coli* and *S. aureus*. Moreover, the nanomaterial–antibiotics combination has successfully inhibited the growth of MDR bacteria in numerous reports. Ag NPs with ciprofloxacin [135], vancomycin [136], and clotrimazole [137] have successfully inhibited the growth of VRE and MRSA species. Au NPs with vancomycin [138] or ampicillin [139] and ZnO NPs with ciprofloxacin [140] also showed antibacterial activity against MRSA and MDR *A. baumannii*, respectively. Therefore, further researches are required for the combination of nanomaterials with antibiotics as this method can be an excellent alternative option for the treatment of infections by MDR pathogenic bacteria.

## 4. Clinical Aspects of Nanomaterials for Antibacterial Activity

Several nanomaterials have been successfully used as “in-use” antibacterial drugs against bacterial infections in humans. These include Ag NPs for burn wound treatment and dental work [38], magnetic NPs for antibiotic drug delivery [22], and SiO_2_ NPs for oral delivery of drugs [23] and AMPs for skin infection treatment [147].

Nanomaterials conjugated with AMPs could be a good strategy to minimize the undesirable features of AMPs such as cytotoxicity, degradation, and inefficiency at the desired target. For instance, Lytixar (LTX-109) and pexiganan are 2 AMPs which are in clinical trials and can be used only for topical use [147]. Another compound, Brilacidin is presently in phase 2 clinical trials for the purpose of curing the infections related to acute bacterial skin and skin-structure infections [148]. A natural AMP, Murepavadin has also been in phase 2 of a clinical trial which inhibits lipopolysaccharide-assembly of protein and can specifically act against *P. aeruginosa*, including its resistant strains [148]. To reduce the problems originating from AMPs, a biodegradable porous silicon NP was used as a delivery vehicle for peptide-based toxin delivery and treatment of *P. aeruginosa* lung infections [147]. With such a conjugation, nanomaterial-AMPs can contribute to the implementation as antibacterial agents to be used in clinical trials. Moreover, there are a plethora of reports, as already discussed in the previous sections, on other nanomaterials or nanomaterial-conjugating agents such as Ag, Au, ZnO, TiO_2_, AMPs, and chitosan regarding the antibacterial activity against MDR pathogens. However, despite the limited clinical trials of nanomaterials for bacterial infections, larger scale clinical trials for all antibacterial nanomaterials are far from reality due to toxicity limitations. Therefore, in spite of its promise, the validity or clinical applications of nanomaterials as potential alternatives to current therapeutics in the treatment of bacterial infections will surely take some time.

## 5. Cytotoxicity of Nanomaterials and Strategies to Tackle

Despite the promising results of nanomaterials for antibacterial activity, some issues still prevail which hinder their application at the clinical scale. There is limited knowledge of nanomaterial interaction with cells and tissues [149]. Therefore, a thorough evaluation and risk assessment for any adverse effects are required before they can be applied as drugs for antibacterial activity. It has been already reported that the intravenously injected NPs can accumulate in bone marrow, liver, lung, colon, and spleen [150]. Moreover, NPs can also enter lung, liver, heart, and brain by inhalation because of their particularly small size and efficient cellular uptake.

There are some additional reports suggesting that the interaction of antibacterial nanomaterial with cells induces free radical-mediated intracellular oxidative stress which may cause hepatotoxicity and pulmonary toxicity [149]. For example, both Ag NPs and Ag^+^ ions induce cytotoxicity to human cells with different mechanisms and higher concentrations [150]. Some nanomaterials also have the ability to increase heart rate, which might be fatal [151]. By considerably increasing the intracellular ROS levels, nanomaterials can also cause oxidative lesions [152]. There are various reports on the biocompatibility studies of different nanomaterials such as ZnO and CuO, where the toxicity generally depends on the size and concentration of nanomaterials and time of treatment [153]. For example, Naskar et al. [96] reported that the Ag–ZnO–graphene nanocomposite is not toxic at low concentrations, but toxic at higher concentrations. However, the nanocomposite itself is a very effective antibacterial material at lower concentration. Thus, there is no need to use the highest concentration which might lead to toxicity. Cha et al. [154] also showed the dose-dependent toxicity of Al NPs where there was no toxicity at low concentrations (5–50 μg/mL), but higher concentrations produced irregular cell shapes and cell shrinkage. Moreover, Li et al. [155] reported that hemolysis in erythrocytes, abnormal sedimentation, and hemagglutination can be caused by NPs. They can also obstruct cytokinesis, chromosome segregation, and centrosome duplication [156]. Sometimes, nanoparticle surface charges also affected the toxicity [149].

Several strategies have already been implemented to tackle the toxicity of nanomaterials. One of the best strategies is to cap the NP with a biocompatible polymer like PEG or chitosan. Various reports have showed that the PEG capping with NPs reduce the toxicity of NPs and enhance the biocompatibility [157,158]. Moreover, these polymers have their own antibacterial activity. Therefore, the capping of NPs with PEG or chitosan not only enhances their biocompatibility, but also synergistically kills bacterial cells with NPs [158,159]. Chia et al. [160] also reported reduced toxicity of ZnO NPs with the help of silica coating. Doping is also another effective strategy to reduce the toxicity of the nanomaterials. In this aspect, Xia et al. [161] reported that Fe-doped ZnO reduced toxicity in the rodent lung and zebrafish embryos by decreasing the dissolution of ZnO NPs. Sekar et al. [162] also reported similar results for the antibacterial activity of Fe-doped ZnO NPs incorporated polyvinyl alcohol nanofibers, which is also biocompatible. Iqbal et al. [163] reported that zinc–silver-doped hydroxyapatite NPs are not only antibacterial, but also biocompatible. Similar results were also reported on graphene film doped with silver NPs [164] and zinc oxide-doped TiO_2_ nanocrystals [165] where the nanocomposites not only showed antibacterial property, but also biocompatibility to mammalian cells. However, it is imperative to say that a systematic investigation is required before any clinical use of nanomaterials as drugs for antibacterial action.

## 6. Limitations of Nanomaterials in Clinical Use

Rapid globalization is one of the prominent causes of increase in MDR bacteria. Despite the advancement of new technologies in the modern scientific world, the discovery of new antibiotics for MDR bacteria is still lagging. The harsh truth in recent times is that the speed at which bacteria cells are acquiring antibiotic resistance is much higher than the discovery of new antibiotics [3,94]. Nanomaterials are promising alternatives to overcome the problems of antibiotic therapies. However, there are two major challenges in the use of nanomaterials for the eradication of MDR bacteria: 1) the limited knowledge of interactions between nanomaterials with human cell [149]; and 2) the control of toxicity from nanomaterials [150,151,152,153] as discussed in a previous section. Moreover, most studies of nanomaterials are generally *in vitro*, therefore they are of no value until and unless the nanomaterials can be tested *in vivo* for antibacterial activity. Factors such as targeted drug delivery, cytotoxicity, stability of nanomaterials inside the body, and leakage information can only be characterized through *in vivo* studies. However, no single method has been developed to identify all above factors associated with the antibacterial activity by nanomaterials. Till now, only some factors affecting the antibacterial activity by nanomaterials have been characterized. These include bacterial strain, action time and concentration-dependent activity. However, even such factors are reliant on the type of used nanomaterials and there is no unified standard for all nanomaterials for antibacterial activity. Moreover, there is no distinctive answer regarding the mechanism of action of nanomaterials against MDR pathogens. Various reports have suggested several antibacterial mechanisms by nanomaterials. These include either single or both ROS generation, which is regarded as the primary mechanism for antibacterial action, and oxidative stress by NPs, depending on the type of nanomaterials [12,29]. However, it will be worthwhile to address the exact antibacterial mechanisms of individual nanomaterials using high-throughput studies. Such mechanisms include intracellular inhibitory mechanisms, oxidative stress of nanomaterials with protein synthesis, gene expression, and metabolism of bacterial cells.

## 7. Conclusion and Future Perspectives

Antibiotics have served as efficient agents to combat several life-threatening infectious diseases targeting humans and have saved many human lives. Since their discovery, antibiotics have always been regarded as the primary treatment line for various bacterial infections. However, this situation is rapidly changing because of the innate bacterial immune resistance to dodge the effect of antibiotics. This is already a global burden on human health which needs immediate intervention. Moreover, the alarming reality is that the emergence rate of new MDR pathogens is much faster than the development of new antibiotics. Pharmaceutical companies have been trying to develop more powerful antibiotics for short-term solutions. However, bacteria are now gaining rapid and strong resistance to even those drugs. In this regard, nanomaterials are currently being heavily researched for their favorable physiochemical properties to tackle the MDR bacteria. In this review, we have specially focused on different types of nanomaterials such as metallic NPs and organic NPs. NPs such as Ag and Au NPs can be potentially used for clinical applications. Graphene, BP, CNTs, chitosan, and AMPs also have the potential to fight against bacterial infections. In addition, we also discussed various new strategies that researchers have used to kill bacteria by using nanomaterials. Furthermore, we introduce the advantages of nanomaterials as therapeutic agents and drug carriers for antibacterial activity. Strategies to tackle the toxicity of nanomaterials for clinical use and some limitations for their use in nanomedicine are discussed systematically. This field of nanomaterial-based antibacterial activity is relatively new compared to the nanomaterial-based cancer studies. Especially, the clinical research data on nanomaterial-based antibacterial activity are very few. Therefore, it requires more in-depth research, including *in vivo* studies for successful transformation of nanomaterials to clinical applications in tackling MDR bacteria.

## Figures and Tables

**Table 1:** Advantages of nanomaterials (NPs) for antibacterial activity.

Multiple Target Approach of Nanomaterials for Antibacterial Activity	Advantages of Nanomaterials as Antibacterial Drug Delivery Vehicle
Reactive oxygen species (ROS) generation	Controllable size of the nanomaterials helps to design targetable antibiotics
Direct interaction of nanomaterial with bacterial cell wall	Drug retention time in blood could be improved
Disruption of bacterial cell membrane	Surface chemistry of NP enables it to be soluble in blood stream
Inhibition of DNA replication and protein production	Nanomaterials can protect antibiotics from detrimental chemical reactions and resistance including opsonization
Inhibition of biofilm formation	Nanomaterials also help the antibiotics to minimize side effects

**Table 2:** Nanomaterial-mediated photothermal effects on antibacterial activity.

Nanomaterials	Target Bacteria	Reference
Ag/ZnO/rGO	*E. coli*	[129]
rGO–Fe_3_O_4_–Au–Ag–Au	*E. coli*	[122]
rGO/Au	*E. coli*, *S. aureus*	[123]
GO–IO–Ag	*S. aureus*	[124]
Fe_3_O_4_–CNT–PNIPAM	*E. coli*, *S. aureus*	[125]
Ag@BP	MRSA	[101]
BP@TiO_2_	*E. coli*, *S. aureus*	[103]
Au@SiO_2_	*E. faecalis*	[126]
Au nanostar	MRSA	[127]
Au NP–IgG	MRSA	[128]
Van–Fe_3_O_4_@Au	PDR *A. baumannii*, *Streptococcus pyogenes*	[57]
GO–IO–Chitosan	*E. coli*, *S. aureus*	[130]

rGO, reduced graphene oxide; IO, iron oxide; PNIPAM, poly(N-isopropylacrylamide); PDR, pan-drug resistant; Van, vancomycin.

**Table 3:** Antibacterial activity of nanomaterials combined with antibiotics.

Nanomaterials	Antibiotics	Target Bacteria	References
Ag NPs	Ciprofloxacin	VRE	[135]
	Vancomycin	MRSA	[136]
	Clotrimazole	MRSA, *S. aureus*	[137]
Au NPs	Vancomycin	MRSA	[138]
	Ampicillin	MRSA, *P. aeruginosa*, *Enterobacter aerogenes*, *E. coli*	[139]
ZnO NPs	Ciprofloxacin, ceftazidime	MDR *A. baumannii*	[140]
Fe_3_O_4_ NPs	Ampicillin	*S. aureus*	[141]
	Ampicillin	*E. coli*, *P. aeruginosa*, MRSA	[142]
CuO NPs	Cephalexin	*E. coli*	[143]
SWCNTs	Ciprofloxacin	*S. aureus*, *P. aeruginosa*, *E. coli*	[144]
GO	Lincomycin hydrochloride Chloramphenicol Gentamycin sulfate	*E. coli*, *S. aureus*	[134]
AMPs-NPs	Gentamicin, vancomycin, azithromycin, amoxicillin	*E. coli*, *S. aureus*, *P. aeruginosa*, *A. baumannii*	[145]
Chitosan	Streptomycin	*Listeria monocytogenes*	[146]
	Ciprofloxacin	Uropathogenic *E. coli*	[133]

SWCNTs, single-walled carbon nanotubes.
